# Supplementary material for: Effective immunity and second waves: a dynamic causal modelling study
Source: Wellcome Open Res. 2020 Sep 30;5:204. Originally published 2020 Sep 1. [Version 2] doi: 10.12688/wellcomeopenres.16253.2 (PMC7549178; doi:10.12688/wellcomeopenres.16253.2)
Supplement: Peer review history

# Peer review: Reviewer response for version 2

**DOI:** 10.21956/wellcomeopenres.17961.r40671

**Published:** 2020-10-09

**Authors:** Anotida Madzvamuse

**Affiliations:** 1 School of Mathematical and Physical Sciences, Department of Mathematics, University of Sussex, Brighton, UK

The authors have addressed fully my comments and happy to approve the revised version. 

 I would recommend the authors to consider referencing this archived report (entirely as optional by the authors as they see fit) since it also sets similar approaches for data-driven parameter values for models for COVID-19 with forecasting capabilities. Predicting and forecasting the impact of local resurgence and outbreaks of COVID-19: Use of SEIR-D quantitative epidemiological modelling for healthcare demand and capacity (2020) ^1^.

Is the rationale for developing the new method (or application) clearly explained?

Yes

Is the description of the method technically sound?

Partly

Are the conclusions about the method and its performance adequately supported by the findings presented in the article?

Partly

If any results are presented, are all the source data underlying the results available to ensure full reproducibility?

Partly

Are sufficient details provided to allow replication of the method development and its use by others?

Partly

Reviewer Expertise:

Mathematical and Computational Biology, Analysis of epidemiological models, Inverse problems, Bayesian Approach.

I confirm that I have read this submission and believe that I have an appropriate level of expertise to confirm that it is of an acceptable scientific standard.

## References

[1] : Predicting and forecasting the impact of local resurgence and outbreaks of COVID-19: Use of SEIR-D quantitative epidemiological modelling for healthcare demand and capacity. *medRxiv*.2020; 10.1101/2020.07.29.20164566 10.1101/2020.07.29.20164566 PMC840786634244764

# Peer review: Reviewer response for version 1

**DOI:** 10.21956/wellcomeopenres.17854.r40237

**Published:** 2020-09-17

**Authors:** Anotida Madzvamuse

**Affiliations:** 1 School of Mathematical and Physical Sciences, Department of Mathematics, University of Sussex, Brighton, UK

The authors present a dynamic causal model to study the quantify the effects and impact of the rate at which effective immunity to COVID-19 is lost. The study follows several works by the authors where some of the details involving the model and the code are described. I found the study lacking key aspects associated with rigorous scientific validation of the model output and its predictions.

1. The dynamical causal model is not fully described but that the reader is referred to previous work by the authors. This is a crucial part of this study, instead the model in its full glory should have been included as supplementary material to aid readability of the work.
2. The study fails to convince the reviewer that a rigorous mathematical/statistical validation has been carried out to understand predictive power of the dynamical causal model.
3. The study also does not present sensitivity of the parameters to understand which parameters are more sensitive to small changes in data.
4. Given that the authors employ the Bayesian approach with detailed datasets, the 25 parameters driving the model should be inferred based on how best the model fits to data. This is not apparent in the study.
5. To really demonstrate the predicting and forecasting power of the methodology, the authors should have taken a subset of the data sets, say, in Figure 3 for example, take data for 20, 40, 60 and 80 days to infer model parameters and use these in the model to demonstrate how accurate they can predict data-points which are known but are in the future. At the moment, predictions are made where it is impossible to compare with data since there is no such data (after say, 100 days). This is a major weakness of the forecasting approach adopted in this work.
6. The authors state that there are many mechanisms that could contribute to the loss immunity and give some examples. However, they are not able to differentiate which ones are key mechanisms. Given the use of Bayesian approach, they should be able to use data to select the best mechanism such the model with such a mechanism best-fits data.
7. In Figure 5, it is extremely difficult to differentiate between model solutions given that the same color is used throughout.
8. The timescale for loss of immunity is prescribed a priori, the authors really could have exploited the inverse approach associated with Bayes theorem to infer this timescale.
9. It is not clear how the effective reproduction number Rt is computed under this proposed approach.
10. It is also not clear if the same graphs are obtained for all the countries included in the study. Does it mean that country-specific characteristics are not important in determining the rate at which immunity is lost?
11. Is Rt the same for all countries?
12. The comparison between the UK and Germany does not seem to make much sense since Germany has had some of the fewest infections due to COVID.
13.  In this study, the authors ignore patient specifics, such as age, ethnicity, location, etc. How important are these factors?

Is the rationale for developing the new method (or application) clearly explained?

Yes

Is the description of the method technically sound?

Partly

Are the conclusions about the method and its performance adequately supported by the findings presented in the article?

Partly

If any results are presented, are all the source data underlying the results available to ensure full reproducibility?

Partly

Are sufficient details provided to allow replication of the method development and its use by others?

Partly

Reviewer Expertise:

Mathematical and Computational Biology, Analysis of epidemiological models, Inverse problems, Bayesian Approach.

I confirm that I have read this submission and believe that I have an appropriate level of expertise to state that I do not consider it to be of an acceptable scientific standard, for reasons outlined above.

# Response

**Published:** 2020-09-21

**Authors:** Adeel Razi

**Affiliations:** Monash University, Australia

Dear Prof. Madzvamuse,

We would like to thank you for the time and effort you have spent reviewing our manuscript. Below are the replies to the comments. We hope these revisions are what you had in mind.

*The authors present a dynamic causal model to study the quantify the effects and impact of the rate at which effective immunity to COVID-19 is lost. The study follows several works by the authors where some of the details involving the model and the code are described. I found the study lacking key aspects associated with rigorous scientific validation of the model output and its predictions.*

*1.           The dynamical causal model is not fully described but that the reader is referred to previous work by the authors. This is a crucial part of this study, instead the model in its full glory should have been included as supplementary material to aid readability of the work.*

*2.           The study fails to convince the reviewer that a rigorous mathematical/statistical validation has been carried out to understand predictive power of the dynamical causal model.*

*3.           The study also does not present sensitivity of the parameters to understand which parameters are more sensitive to small changes in data.*

*4.           Given that the authors employ the Bayesian approach with detailed datasets, the 25 parameters driving the model should be inferred based on how best the model fits to data. This is not apparent in the study.*

*5.           To really demonstrate the predicting and forecasting power of the methodology, the authors should have taken a subset of the data sets, say, in Figure 3 for example, take data for 20, 40, 60 and 80 days to infer model parameters and use these in the model to demonstrate how accurate they can predict data-points which are known but are in the future. At the moment, predictions are made where it is impossible to compare with data since there is no such data (after say, 100 days). This is a major weakness of the forecasting approach adopted in this work.*

Many thanks for these comments and critiques. These are understandable points that we hope to have addressed by delimiting the scope and target of this report. For example, this is not an example of forecasting – it was meant as a worked example of Bayesian model comparison to get at a key model parameter. We have included the following qualifications in the new Discussion section and also updated the Background section in the revised manuscript – but would be happy to revisit or extend this discussion if the reviewer thought appropriate.

- "As noted in the introduction, this technical report should be read as part of a series demonstrating the application of variational Bayesian inference to quantitative epidemiological modelling. The first, foundational report (Friston *et al.*, 2020d) established the nature of the model and attending inversion scheme. The second dealt with coupling models of a single region or population to illustrate how exchange between populations can be handled (Friston *et al.*, 2020b). The third report (Friston *et al.*, 2020c) focused on posterior predictions and projections under different scenarios (e.g., testing and tracing). This report illustrates a particular application of Bayesian model comparison (strictly speaking, Bayesian model reduction) known in some fields as structure learning (Friston *et al.*, 2016). As such, the estimates and inferences reported here should not be taken as definitive. Rather, we have described the procedures that could be used to furnish these kinds of estimates during the course of the current epidemic or in the future."

- "One may ask why we chose to use Bayesian model comparison to form posterior beliefs about a particular parameter of the model, as opposed to simply evaluating its posterior under the Laplace (i.e., Gaussian form) assumption? Our motivation was twofold. First, it shows how one can eschew the Laplace assumption and use Bayesian model reduction to build a non-Gaussian posterior belief over a parameter of interest. For example, the posterior could have been bimodal. This application of Bayesian model comparison shows how it is possible to leverage the computational efficiency of variational Bayes, without committing to a fixed-form posterior over one or a small number of interesting parameters. The second reason—for illustrating Bayesian model comparison in this way—was to show how to accumulate evidence from multiple datasets (here, different countries). This pooling reduces to adding the (logarithms of) evidence for the same model from independent data. Note that a model is defined here in terms of prior beliefs. This means that one can use Bayesian model reduction to score the quality of any prior beliefs empirically. In one sense, this is an example of empirical Bayes (Efron and Morris, 1973; Kass and Steffey, 1989; Friston *et al.*, 2016)."

- "Although our focus is on Bayesian model comparison, it may be useful to rehearse the distinction between the variational approaches used in dynamic causal modelling and the usual approaches found in the epidemiological literature. Perhaps the most important difference is the way that model evidence or marginal likelihood is handled or evaluated. In variational Bayes, this is computed explicitly in terms of an evidence bound afforded by the variational free energy (Beal, 2003; Winn and Bishop, 2005; Fox and Roberts, 2011). This uses the entire time series and a computationally efficient scheme afforded by assumptions about the shape and factorisation of an approximate posterior. The alternative would be to eschew any assumptions about the form of the posterior and approximate the marginal likelihood of a model in terms of its crossvalidation accuracy. Technically, the log of model evidence is accuracy minus complexity. In the setting of crossvalidation, one can ignore the complexity term and approximate model evidence with the accuracy with which some new (i.e. test) data are explained (MacKay, 2003). On this view, crossvalidation accuracy becomes another approximation to log evidence. Models with a greater evidence are those that, on average, generalise to new data and therefore have the greatest predictive validity. This means that variational approaches evaluate model evidence or marginal likelihood explicitly, while other approaches (e.g. stochastic or sampling approaches) use crossvalidation or predictive validity. Although it is possible to demonstrate the predictive validity of dynamic causal models by withholding test data—see Figure 13 in (Friston *et al.*, 2020d)—this is not necessary because we already know that model selected has a greater evidence than another model."

- "In a similar vein, the conventional epidemiological modelling literature often features sensitivity analyses of the parameters. For a sensitivity analysis of the dynamic causal model used above, please see Figure 9 in (Friston *et al.*, 2020d). These kinds of analyses allow one to eyeball which parameters make a difference or, from the point of view of model inversion, which parameters are informed by the data. Although a useful adjunct to dynamic causal modelling, sensitivity analyses of this sort are not necessary to understand the relationship between model parameters and data. This is because the sensitivity (i.e., the derivative of the data with respect to model parameters) is an integral part of the posterior uncertainty under the Laplace assumption: see Equation 23 in (Friston *et al.*, 2007). In other words, the sensitivity is handled implicitly in terms of uncertainty quantification or the posterior credible intervals ascribed to various parameters. In brief, a parameter that has tight credible interval implies that small changes in this parameter produce large changes in data space. The ensuing estimates of posterior uncertainty are, effectively, then used to marginalise out uncertainty about the parameters to furnish the marginal likelihood required for model comparison."

- "Note that the posterior maximises model evidence, which is the same as maximising accuracy while minimising complexity. Complexity in variational inference corresponds to the Kullback-Leibler divergence between the prior and posterior (Penny *et al.*, 2004). Heuristically, this can be regarded as the degrees of freedom used up to explain the data. Crucially, this means that the data fit or accuracy is only half the story. One has to provide an accurate account of the data as simply as possible. Procedures based upon the Akaike and Bayesian information criteria do not evaluate the complexity explicitly and can be dangerously misleading when used for model comparison (Cornish and Littenberg, 2007; Penny, 2012). This speaks again to the potential utility of variational procedures in epidemiological modelling."

It was useful to have your perspective on what constitutes a rigorous statistical validation and we hope to have addressed your concerns above. Highlighting what variational (Bayes) procedures bring to the table (including a much simpler and easier way of doing things) is the *raison d'être *of these reports. 

*6.           The authors state that there are many mechanisms that could contribute to the loss immunity and give some examples. However, they are not able to differentiate which ones are key mechanisms. Given the use of Bayesian approach, they should be able to use data to select the best mechanism such the model with such a mechanism best-fits data.*

This is a very good point. We have included the following in the Background section to address it:

- "In talking about a loss of effective immunity, we assume that there could be many contributions to this loss. For example, a decline in antibody levels, viral mutation, or a dilution of population immunity due to population fluxes. These are all important mechanistic hypotheses that can, in principle, be addressed using Bayesian model comparison. To do this, it would be necessary to parameterise the model in a way that allowed one to withdraw one or other mechanism and evaluate whether the model evidence increased or decreased. An example of this can be found in (Friston *et al.*, 2020a), where the relative contribution of lockdown and population immunity to prevalence and mortality was evaluated. Interestingly, both lockdown and herd immunity were necessary to explain the data: in the sense that removing either mechanism substantially reduced model evidence. Please see (Friston *et al.*, 2020a) for details." 

*7.           In Figure 5, it is extremely difficult to differentiate between model solutions given that the same color is used throughout.*

Thank you – we have adjusted the colour scheme.

*8.           The timescale for loss of immunity is prescribed a priori, the authors really could have exploited the inverse approach associated with Bayes theorem to infer this timescale.*

It would certainly have been possible to report the Gaussian posterior following a model inversion under uninformative priors. However, as noted above, this would commit to a Gaussian form for the posterior. Furthermore, this would involve a slightly more complicated approach to assimilating the data from multiple countries (using Bayesian parameter averaging). We could go into this in more detail if you thought it necessary.

*9.           It is not clear how the effective reproduction number Rt is computed under this proposed approach.*

The effective reproduction number is computed as described in (Friston *et al.*, 2020c). We have now included this in the revised manuscript (in the Results section) as follows:

 

- "The effective reproduction rate provides a useful statistic that reflects the exponential growth of the prevalence of infection. There are several ways in which it can be estimated. For our purposes, we can evaluate an instantaneous reproduction rate directly from the time varying prevalence of infection as follows: (Mathematical typesetting feature is not available in this Editor. Please refer to the Results section in the revised draft.) These expressions show that the reproduction rate reflects the growth of the (logarithm of the) proportion of people infected—and the period of being infectious. This is related to the doubling time *T _d_*. Note that the reproduction rate is not a parameter of the model: it is an outcome that is generated by the latent states inferred by inverting (i.e., fitting) the model to empirical timeseries."

 

*10.         It is also not clear if the same graphs are obtained for all the countries included in the study. Does it mean that country-specific characteristics are not important in determining the rate at which immunity is lost?*

This is a very good question. We have tried to answer it – as a footnote 1 in the revised draft – with the following:

- "By pooling the evidence over countries in this way, we have allowed for country-specific differences in the parameters shaping their epidemics. However, we then assume that all countries share the same loss of immunity. A consideration of between country variations and conditional dependencies between parameters would require a different approach. Usually, this would be addressed using hierarchical Bayesian models (a.k.a., parametric empirical Bayes). An example of this can be found in the first report examining within and between country differences: see Figure 6 in (Friston *et al.*, 2020d)." 

*11.         Is Rt the same for all countries?*

No. It is evaluated at each point in time on the basis of latent states that are generated from the model parameters (please see above). These parameters were estimated independently for each country. The model evidence under different priors about the immunity parameter is then pooled over countries. This can be regarded as a form of empirical Bayes (please see above).

*12.         The comparison between the UK and Germany does not seem to make much sense since Germany has had some of the fewest infections due to COVID.*

This question reflects prior assumptions about the relationship between the prevalence of infection at the onset of the outbreak and the occurrence of a second wave. It is precisely these prior assumptions that the current modelling allows one to address. The nonlinearity in these models can sometimes confound prior assumptions of the sort.

*13.         In this study, the authors ignore patient specifics, such as age, ethnicity, location, etc. How important are these factors?*

It is likely that they are very important. Again, questions of this sort can only be answered definitively by including models with the patient specifics in them. We have added the following in the new Discussion section where we discussed limitations and future work:

- "In our treatment of heterogeneity, we have not explicitly modelled things like age, ethnicity, population density etc. Instead, we have simply modelled the implicit heterogeneity—at a coarse-grained level—by using a series of bipartitions of the latent states. More refined models could consider stratification by age or ethnicity, with appropriate contact matrices. Whether this fine graining of heterogeneity is justified by the data can be cast as a question of model comparison. If the model is too expressive or complex for the data at hand, including age stratification will reduce model evidence. However, with disaggregated and sufficiently long time series, Bayesian model comparison could, in principle, identify whether the attributes above play an important role. And, if they do, one could assess their quantitative contribution in terms of the posterior parameter estimates."

 

**References:**

Beal MJ. Variational Algorithms for Approximate Bayesian Inference. PhD Thesis, University College London 2003.

Cornish NJ, Littenberg TB. Tests of Bayesian model selection techniques for gravitational wave astronomy. Physical Review D 2007; 76(8).

Efron B, Morris C. Stein's estimation rule and its competitors – an empirical Bayes approach. J Am Stats Assoc 1973; 68: 117-30.

Fox C, Roberts S. A tutorial on variational Bayes. Artificial Intelligence Review 2011; 38(2): 1-11.

Friston K, Costello A, Pillay D. Dark matter, second waves and epidemiological modelling. medRxiv 2020a: 2020.09.01.20185876.

Friston K, Mattout J, Trujillo-Barreto N, Ashburner J, Penny W. Variational free energy and the Laplace approximation. NeuroImage 2007; 34(1): 220-34.

Friston K, Parr T, Zeidman P, Razi A, Flandin G, Daunizeau J *, et al.* Second waves, social distancing, and the spread of COVID-19 across America [version 1; peer review: awaiting peer review]. Wellcome Open Research 2020b; 5(103).

Friston K, Parr T, Zeidman P, Razi A, Flandin G, Daunizeau J *, et al.* Testing and tracking in the UK: A dynamic causal modelling study [version 1; peer review: awaiting peer review]. Wellcome Open Research 2020c; 5(144).

Friston KJ, Litvak V, Oswal A, Razi A, Stephan KE, van Wijk BCM *, et al.* Bayesian model reduction and empirical Bayes for group (DCM) studies. Neuroimage 2016; 128: 413-31.

Friston KJ, Parr T, Zeidman P, Razi A, Flandin G, Daunizeau J *, et al.* Dynamic causal modelling of COVID-19. Wellcome Open Research 2020d; 5(89): 89.

Kass RE, Steffey D. Approximate Bayesian inference in conditionally independent hierarchical models (parametric empirical Bayes models). J Am Stat Assoc 1989; 407: 717-26.

MacKay DJC. Information Theory, Inference and Learning Algorithms. Cambridge: Cambridge University Press; 2003.

Penny WD. Comparing dynamic causal models using AIC, BIC and free energy. Neuroimage 2012; 59(1): 319-30.

Penny WD, Stephan KE, Mechelli A, Friston KJ. Comparing dynamic causal models. Neuroimage 2004; 22(3): 1157-72.

Winn J, Bishop CM. Variational message passing. Journal of Machine Learning Research 2005; 6: 661-94.

# Peer review: Reviewer response for version 1

**DOI:** 10.21956/wellcomeopenres.17854.r40238

**Published:** 2020-09-15

**Authors:** Michael Stern

**Affiliations:** 1 Laboratory of Cardiovascular Science, Intramural Research Program, National Institute on Aging, National Institutes of Health, Baltimore, MD, USA

This article uses a version of the established LIST model to attempt to estimate the rate of decay of immunity of COVID-19 from published mortality data from a number of countries. It finds that the most probable immunity lifetime is 3 months. While perfectly consistent with present evidence about other coronaviruses, this is an alarming finding. Several topics require discussion, particularly possible limitations that might allow escape from this dire prophecy.

1. This was done by a novel method, running multiple models for a range prior values of the immune lifetime, and comparing pooled model evidence among models. Why was this done? If the signal for early decay of immunity is as strong as indicated, I would have thought it would come out by simply floating the decay rate as one of the many parameters and estimating its posterior in the fit.
2. Obviously, any signal about the decay of immunity must derive from the slope of the decaying phase of the infection wave. There are a variety of ways this could be misleading. While a simple form of heterogeneity is included in the form of sub-populations that are resistant, unable to transmit or effectively isolated, events and antibody data since this paper was submitted have shown, at least in the US which unfortunately dominates mortality data, that there is extreme heterogeneity of the infection on almost every spatial scale, down to the single neighborhood in New York City. These local waves of infection are no longer synchronous, if they ever were, and would be expected to put a "tail" on the first wave that might be hard to distinguish from the decay of immunity. Experience in nursing homes and prisons has shown that it is extremely difficult to have any subgroup that is truly isolated from the epidemic, and those areas again may respond late but still make a major contribution (e.g. ~45% of US mortality). In the absence of age stratification, mortality data from this virus is likely to be highly biased towards groups whose connection to the rest of society may be weaker (and therefore slower) than average.
3. The model uses an internal feedback loop to effectively estimate how social distancing interventions respond to epidemic numbers. This may be applicable in some societies, but the libertarian ethos (and then politicization) in the US has resulting in "re-opening" that is both chaotic in time and heterogeneous geographically. As the authors acknowledge, premature relaxation of mitigations can be conflated with loss of immunity. Experience since the end of the authors' data sets has shown new waves of infection related to relaxation of controls well in advance of the predicted time of the "second wave" estimated using the simple, exponential decay of effective immunity. Indeed, in many areas, the (apparent) number of infections has formed plateaus that are hard to account for by any homogeneous, compartmental model.
4. A naive, technical question for my own understanding about the underlying model structure: From the earlier papers, I gather that the likelihood of outcome variables is computed as a product of independent binomials (approximated by gaussians) over time, i.e. the only uncertainty is from sampling error in determining the individual counts at each time point. Direct stochastic simulation would predict that outcome points would be correlated, e.g. more cases now predicts more deaths after a lag. Granted that this likelihood is supposed to be marginal over the (unknown) distributions of parameters and latent compartments, is that sufficient to justify the independence assumption? The structure of the transition matrix itself would seem to imply temporal correlations. 

Is the rationale for developing the new method (or application) clearly explained?

Yes

Is the description of the method technically sound?

Yes

Are the conclusions about the method and its performance adequately supported by the findings presented in the article?

Yes

If any results are presented, are all the source data underlying the results available to ensure full reproducibility?

Yes

Are sufficient details provided to allow replication of the method development and its use by others?

Yes

Reviewer Expertise:

Stochastic simulation and propagation, usually applied cardiac calcium signaling, but lately to simulation of the ongoing coronavirus pandemic.

I confirm that I have read this submission and believe that I have an appropriate level of expertise to confirm that it is of an acceptable scientific standard.
